# American Dermatological Association

**Published:** 1882-12

**Authors:** 


### Society Reports.

ARTICLE II.

AMERICAN DERMATOLOGICAL ASSOCIATION. (Continued from page 513, November number).

### SECOND DAY—THURSDAY, AUG. 31.

### MORNING SESSION.

The President called the meeting to order at ten o'clock. Dr. W. A. Hardaway, of St. Louis, reported a case of Pigmented Neoplasm of the Skin, and presented a colored portrait of the patient for inspection.

### DISCUSSION.

Dr. Heitzmann, being called upon for his report of the microscopical appearance of the growth, said that he had received a small piece of the tumor, about one-fourth of an inch in diameter, which he had carefully examined. A transverse section had one striking peculiarity to the naked eye; that is, the linear portion of the growth, close above the subcutaneous tissue, contained dark brown pigment, while those parts above were spotted. Microscopically, the epidermal layer was considerably thinned, consisting only of three or four rows of cuboidal cells, beside the usual epithelium. The Malpighian layer was nearly lost; the lower layer of epidermis was nearly normal. The derma proper, on the contrary, was considerably altered, and greatly thickened; in this thickened portion several things were noticed. Growing toward the subcutaneous tissue were nests filled with tissue constituted entirely of medullary and embryonal bodies; there were some structureless bodies, corpuscles with indistinct nuclei, and others with distinct nuclei, the detection of which required high powers. Lastly, there were bodies among the mass in the nests, in which plastids could be distinctly made out by studying the nuclei; to these the term myéloplaxes has been applied. In all this there were none of the characteristics of cancer; no marked reticulated body; no epithelial tissue, or nests of nucleated cells; but the material was evidently to a great extent newly formed. In this growth we find pigment accumulating in the medullary tissue in the globular nests, and sparsely in the connective tissue around the nests. The red blood-globules are unchanged, but the delicate reticular tissue is entirely filled with dark pigment.

It follows from this description, that the tumor was a new formation; and the existence of nests entirely devoid of basis substance to constitute medullary tissue, indicates a considerable degree of malignancy. But it is not cancer, for here the epithelial layers were diseased. Therefore, there only remains one variety to consider; it is a sarcoma, or, as he prefers to call it, a myeloma. Three varieties, round, spindle, or giant-cells, are usually described, and several additional forms have been distinguished. Those containing pigment have been called melanotic sarcoma. There is also the angio-lethic sarcoma, or psammoma of Virchow; and Billroth had another variety, which he termed medullary sarcoma, containing alveoli filled with cells (but no epithelium); this was accepted by Cornil and Ranvier, but Billroth in later years gave up the diagnosis of alveolar sarcoma, and concluded that it is a highly developed cancer. He (Dr. Heitzmann) had seen, both in Vienna and in New York, a number of these cases which appeared originally in the skin. One case, seen five or six years ago, he recalled.

The tumor was in the left groin. It formed a small nodule something like a mole. It was extirpated, but recurred; repeated extirpations ended in a like result. After a year or two, the tumor grew very rapidly, and was the size of a child's head. The consulting physicians pronounced it cancer. The overlying skin was so thin that they anticipated ulceration. Shortly afterward, the patient began to vomit a large quantity of sputa, without exhibiting much bronchitis. He (Dr. Heitzmann) examined the sputa, and found, beside the ordinary mucus and pus corpuscles indicative of bronchitis, a large number of globules, smaller than pus elements; in fact, medullary corpuscles indicating myeloma, and predicted that the secondary formations were forming in the lungs. From this, also, he stated that the primary tumor was not cancer, but really sarcoma, simply from the examination of the sputa. Two months later the patient died, and the lungs were found studded with these sarcomatous granulations; the tumor was also found to be a medullary sarcoma.

The second case occurred in the skin of the large toe in a patient living in the country. The toe was amputated, and an examination revealed the fact that the growth was a very malignant type of alveolar sarcoma.

The third case was this one of Dr. Hardaway's, in speaking of which he said, "I adhere to this diagnosis of alveolar sarcoma, although Billroth has abandoned it, because I think that a tumor which gives rise to secondary tumors which are sarcomatous, must be sarcoma originally. The appearance of the growth in this case described by Dr. Hardaway, showed that at first it was not of a sarcomatous type, but afterward took on these characteristics, which are those of the worst form of sarcoma—a melanoma or pigmented sarcoma. The loose pigment in the center indicates that it has been an unpigmented growth covering over a pigmented one; now, I declare it to be a pigmented medullary sarcoma, the most malignant form of sarcoma. Such tumors generally occur in the skin of the hands and feet; they usually end within two years from the start. I have seen two or three cases in New York. Mr. D. had brown spots which appeared in his hands; afterward nodules formed, and I was called in consultation. After a microscopic examination, I predicted death within three years He died two months after the three years were up."

This case reported by Dr. Hardaway is not one of the worst instances, because originally the growth was local, and there is a tendency to involution, which is not uncommon in myeloma. Swelling of the lymphatics has nothing to do with the tumors. The prognosis is death within three or five years, with the formation of sarcoma nodules in the internal organs.

Dr. Duhring said that during the reading of the notes of the case by Dr. Hardaway, but especially toward the close, where the lesions were described, he had been struck with a certain resemblance to a case he had brought before the Association two years ago. This was omitted in discussing the general diagnosis, and the speaker would like to hear the lecturer's views. On looking at the portrait, he could not help noticing the resemblance to the patient he had presented. It had the same features, especially the orange-colored border ; apparently the same lesions, occurring on the body, shoulders, and on the neck and breast, which seemed to the speaker to be almost identical in their clinical aspect with the case now under discussion. He therefore had been surprised by the remarks of Dr. Heitzmann as to the conclusions he had drawn with regard to the nature of the disease. The pigmentation is not brought out very well in the portrait, being simply an obscure discoloration, but the color as seen in the border is almost identical with the case previously brought before the Association. Moreover, the tumor over the nose between the eyebrows is most suggestive of the former case. He asked, with regard to the pigmentation, whether the portrait was reliable ?

Dr. Hardaway said that the portrait was very accurate. The centers are entirely free from pigment, but the border is darkly pigmented, with an orange color, except in the primary spots where the pigmentation is very decided.

Dr. Robinson remarked that if it is an alveolar sarcoma, it is strange that it had lasted so long. He asked if it is usual for an alveolar sarcoma to last eight or ten years ?

Dr. Heitzmann stated that the tumor originally started with very little malignancy about it. The cells of the older portion were comparatively small ; it was only the growth of the last year which showed the larger nests, and consequently a more malignant type.

Dr. Robinson had generally regarded round-celled tumors as being rather rapid in their growth.

Dr. Heitzmann said that it is true, as the rule. Dr. Duhring's case was a round-celled sarcoma, but developed slowly.

Dr. Taylor asked of Dr. Duhring if he would admit the diagnosis of sarcoma in that case, which he presented before the Association as an inflammatory fungoid neoplasm ?

Dr. Duhring replied that he had accepted this view.

Dr. Taylor said that he asked the question because he would like that statement to go upon the record.

Dr. Atkinson reported the following case: An old German, a Hebrew, had within the last few years developed a very unusual condition of the feet. On the left foot, especially, there are a number of small pigmented tumors, looking like little blood-blisters with their contents coagulated, scattered over the foot; there were also large superficial blood-vessels. The lesions evidently began as angioma, and afterward became pigmented, forming melanotic sarcoma. On the leg are several small spots of the same color, resembling the discoloration remaining after chronic eczema in this situation ; but they could have no dependence upon mechanical hyperæmia in this instance. The spots are sharply defined, and are permanent. In connection with the case reported by Dr. Hardaway, he would merely mention one point: that all these cases have relations to each other, although the appearances may vary; he believed that this view would simplify the diagnosis. When Dr. Heitzmann mentioned six forms of sarcoma, he merely referred to types. Nature, however, does not jump from one to another, but presents intermediate forms. Therefore, if we group several general ideas together, it will be found that there are some features in common, a consideration of which will prevent us from being puzzled. The general conclusion that there is this wide limit to the type, and an almost imperceptible merging of one form of new growth into another, will simplify diagnosis very much. Then, again, we have the interesting question as to the co-existence of tumors, probably not dependent upon metastasis, nor upon constitutional causes, which is an old doctrine, but perhaps due to trophic disturbances from causes residing in the nervous system. The fact is, that the new growth depends not upon a constitutional condition, but upon local conditions, which may be wide-spread, for instance in the skin, and which gives rise to similar tumors in different localities. This is also exhibited by some benign tumors, as fibroma molluscum, and certain forms of nævi. This is mentioned in order to show that the constitutional causes are not necessary to explain the wide-spread character of the lesion.

Dr. Piffard said that he understood that the characteristics of this growth were the absence of change in the epithelial layers and the occurrence of lobuli filled with plastids and granular substance, and that this co exists with the deposit of pigment. He stated that he has in his possession sections showing the same appearances: absence of changes in the epithelial layers; lobuli filled with cells. One of these cases he had reported, and Dr. White reviewed the paper, and declared that it was a case of epithelioma, although there was no dipping down of the epithelial layers into the rete. What was remarkable in his case was, that ulceration was so long deferred; in one case it was eight years, in another two years. Friedrich Friedländer and Thoma Coloniati also refer to this peculiarity.

The case he now referred to was one he had described as rodent ulcer, *lupus exedens*, and the condition is also described by the authorities just named, as well as by himself eight years ago. The only difference that he could see was that in his own case, there were pigmented granules; otherwise it was the same, so far as descriptions and drawings are concerned. He did not, however, consider this as agreeing with Dr. Duhring's case, but rather as belonging to rodent ulcers, rather lupoid than epitheliomatous. When it comes to the diagnosis of sarcoma, there is no uniformity in the distinction, and the word sarcoma has been applied to everything which is not carcinoma. If we accept this definition of sarcoma, then the growth is sarcomatous, but it is not so in the ordinary application of the term.

This disorder is not very malignant during the life of the patient; it is a slow growth, and slowly tending to ulceration. The point he wished to make is, that this development tends to enlargement of glands, or has a strumous tendency. He understands that Dr. Fox, who saw the case, also regarded it as of a lupoid nature.

Dr. Hardaway said that Dr. Fox had seen the case, but he was not aware that he had come to a positive conclusion with regard to it.

Dr. Heitzmann said that oncology is a most interesting study, but before our own day such knowledge was not possible. Virchow commenced to write a work on tumors, but never finished it. Ten years have since passed, and the science has made great advance. His work on the Microscopic Morphology of the Animal Body in Health and Disease is now in the hands of the binder, and will be issued next week. In it this question had been discussed more completely than he could do at present. The whole subject of tumors has been much simplified; under twelve heads we can now place all tumors, with several varieties; so that there need be not much difficulty in classifying tumors. Still, there are questions that are not satisfactorily settled; among these is the origin of sarcoma. Dyscrasia, etc., are terms which for hundreds of years have filled medical books, but are still unexplained. Everybody speculates upon these matters, but without much profit; if one contends that the cause is constitutional, he finds support; if he says it is local, he will also find others to agree with him. The stand taken by the speaker is that "we don't know." Let us wait until facts come to throw light upon the etiology of tumors, but so far we do not know, and that closes all discussion.

Regarding Dr. Piffard's remark as to the structure, he must say that these tumors have nothing in common with epithelioma, and nothing in common with lupus. He had called them sarcoma, and the doctor had reminded him that the definition of sarcoma varies from week to week. This really is not true. The histological characters of sarcoma were established twenty years since by Virchow, which, in short, are "embryonal connective tissue and basis substance," which is eminently satisfactory. The fact that there are degrees of malignancy, is no objection to this view. We may tell the degree of malignancy by watching the microscopic characters. The fact is, that the amount of fibrous tissue shows sufficient to account for the slow growth in the cases under consideration; and the thickened appearance of the tumors affords, therefore, a better prognosis. With regard to epithelioma, there is no increase of epithelial tissue; with regard to lupus, it exhibits an entirely different microscopic structure; it is an inflammatory process more or less; certainly lupus has not the slightest resemblance to sarcoma.

Dr. Hyde said that he had been much interested in the cases just reported; and it seemed to him that the proper performance of the work of this Association should be a subject of congratulation among its members, and especially the opportunity of seeing such rare cases of disease as that presented by Dr. Duhring at the last meeting. He had himself had a case which should be classed with these; his misfortune was that the death of the patient only occurred a few days before he left Chicago, and he had not had the time to prepare the sections and report the case for presentation. A piece of the tumor which had been excised, had been examined by Dr. Heitzmann, who pronounced it a case of globo-myeloma or sarcoma. The case is of increased interest since he had reported it, as he had watched the symptoms going on until death, and had taken the time to follow up the case. He had reported the case to the authorities as one of death from sarcoma. It is to be noticed that no special reference was made to the case in the classification in his address of Saturday, except in its proper place under the sarcomata.

Dr. Piffard said that Dr. Heitzmann had referred to the pathology of lupus as if it had been conclusively settled; but if he refers to the usual authorities, he will find that it is very unsettled. He spoke of it as simply a form of inflammation, but this will depend upon what he understands by inflammation. Microscopically, he will find in some a diffuse infiltration of small round cells; in others giant-cells; in some, aggregated in nests, in still others, infiltrating, and associated with a varying amount of inflammation, which is characteristic. We have different forms of lupus, which differ widely; he could not see any connection between those having small round cells, and others containing the giant-cells or myèloplaxes of the French authors, such as are also found in syphilis, phthisis, and other diseases.

Dr. Hardaway said that he must confess himself much interested in the discussion of his case. From a clinical standpoint, he was himself inclined to regard it as a case of lupus, and for this reason adopted the name by which it is announced in the programme, simply terming it a pigmented neoplasm. He noticed the resemblance to Dr. Duhring's case from the written description. He could not understand the prognosis given by Dr. Heitzmann. The case had gone on for ten years; the first infiltrated plaques upon the forehead were in the same condition as they were years ago, except that they appear to be undergoing involution. He promised to report from time to time the future progress of the case, in order to follow up the natural history as far as possible.

Dr. Hyde regretted very much indeed that Dr. Hardaway was absent last year, and also that the speaker himself was not present when Dr. Duhring presented his case. This shows the importance of attending regularly the meetings of the Association. During the discussion of this case of Dr. Hardaway, he had been struck particularly with its resemblance to Dr. Duhring's case.

AFTERNOON SESSION, THREE O'CLOCK.

Dr. Robinson read a paper on The Nerves of the Skin.

Dr. Heitzmann, in opening the discussion, said that it was only a little over a month since Dr. Robinson had shown him these specimens, and when he examined them, he was simply amazed; they furnish the solution of a puzzle, where we had none before. The general opinion as to the termination of the skin-nerves is, that they form bulbs; beside the Pacinian corpuscles, we know that there are end-bulbs, described by Meissner, Wagner, and others; but the actual mode of termination of tactile nerves within the bulbs was not known. Dr. Robinson has settled this question. Our Association must be congratulated upon first listening to such a paper, but a little credit was claimed by the speaker for inducing Dr. Robinson to bring it before the Association. In fact, he could corroborate every word that is contained in this communication. What the lecturer had discovered upset all our previous ideas on the subject. It was formerly supposed that the nerves terminated like buds, and that impressions were conveyed backward from these papillæ to the nerves; but much difficulty existed with regard to the Pacinian corpuscles, which are generally found in the deeper layer of the derma, and in remote situations, like the mesentery, and other situations in which ordinary sensation is not present, as in the periosteum of the petrous portion of the temporal bone.

Before hearing Dr. Robinson's explanation, he could never understand why they existed in these places. We knew that many medullated nerve-fibers end in plexuses, but he regarded the formation of a loop as just as much a termination of a nerve in the case of the Pacinian corpuscles as in the other; the new fact is that they uniformly end in a plexus, and that one nerve enters the bulb, but another always leaves it, and that there is at the point where the bulb exists a plexus; an apparatus for condensing the nervous impression, like the electrical apparatus. He repeated that he would answer for every word that the lecturer had said, and he wished to express his admiration of the paper presented.

Dr. Piffard merely wished to endorse Dr. Heitzmann's remarks, and to state that we are very fortunate in having in the Association those who are able and willing to conduct such researches, which must be a labor of love, and therefore deserve full consideration and appreciation.

Dr. Hyde said that he could only repeat, on the part of all who have heard the admirable paper, their satisfaction and pleasure at witnessing such admirable work.

Dr. Robinson said, in conclusion, that he had hesitated very much before bringing this subject before the Association, for fear that it might not interest the members. He was very glad at its kind reception, and that it was found of any interest whatever. He felt that the truth must come out at some time; it is a duty to ourselves to state the truth, and to look for justice to the future. He invited the members to examine the specimens which he would present in the morning.

Dr. J. C. White, of Boston, read a paper on the Question of Contagion in Leprosy.*

DISCUSSION.

Dr. Rohé said that a few years ago he had reported two cases of leprosy which had come under his observation, and he recalled a third case, not yet reported. The first case was a man, a native of New York, who went to Cuba, and resided there from eight to nine years, and then returned to this country, and lived in Baltimore. A number of years after his return, he thought fourteen, he first manifested symptoms of leprosy. Between two and three years after the symptoms first appeared, he came under Dr.

* Published in the *Amer. Jour. of the Med. Sci.*, Oct., 1882.

Rohé's care. There was no doubt about the disease; it was a typical case of tubercular leprosy. The man was a liar. He gave one history to the speaker in Baltimore, and afterward went to New York, where he gave another history to Dr. Fox; but it is probably true that the period of time which elapsed, from his leaving Cuba, where there were lepers, and the first manifestation of the disease here, was about fourteen years. The patient subsequently died in New York.

While attending this case in the hospital in Baltimore, in 1878, another patient consulted him. A woman, a native of Maryland, had gone in 1855 to New Orleans, where she had yellow fever, but recovered. She subsequently traveled through the South, and returned to Baltimore in 1866. In 1869 she was pregnant with her last child, and while pregnant, the symptoms first manifested themselves, appearing first as large bullæ particularly. This case was published in the *American Journal of the Medical Sciences*, in July, 1878. The woman died in 1878 or 1879. The child which she was carrying when the symptoms first appeared, was a very fair little girl, when seen in 1878. Subsequently, on his return to Baltimore, he had searched for the little girl, and found that she had had scarlet fever, but no evidence of leprosy. The husband of the woman had lived with her and cohabited with her for nine years after the appearance of the leprosy, but exhibited no signs of the disease himself, and no member of her family had shown any of its manifestations. Dr. Rohe believed that for a time the man Brown, the first case, had lived in the same section of the city, but how near, he had not been able to discover. Of the diagnosis there can be no doubt; Dr. Atkinson also saw the cases. They were both typical cases.

In 1879 he had visited New Orleans, and saw a case afterward published in the Board of Health Reports of Louisiana, coming from Tèche parish, one of the infected parishes. A point of interest in the history of the disease occurs in this connection. Among the alleged origins of leprosy in Tracadie, is stated to be the arrival of some creoles from Louisiana, and among them was the family of a Frenchman from St. Malo, in France. The first case that occurred in Louisiana was that of a Catholic priest who had been ministering to these patients in the Tèche parishes; he was never confined in a hospital, but he was seen by Dr. Brown, who pronounced it a case of leprosy, and he died within a month.

As to inoculation, he found it hard to believe that a disease like leprosy could be inoculated; and the cause assigned by the doctor in the Sandwich Islands, the pricking of a pin, is very doubtful. Such slight lesions are so frequent, and where the period of incubation extends over a year, there may be a question as to the exact time and manner of inoculation. So far as the bacillus lepræ is concerned, he could not offer an opinion from personal observation. The practical objection to the bacillus theory, is the difficulty in seeing any likeness between leprosy and the ordinary contagious diseases. The period of incubation varies from one to sixteen years; no other disease shows such variation. Then, again, we must admit inoculation as the principal method of communication, but he had not been able to satisfy himself that leprosy is inoculable in the ordinary sense. That it may be inoculated is possible, but the only experiment that could settle this point must be made upon human beings. As far as the danger from this course is concerned, he would be willing to be inoculated with the bacillus lepræ, if he were willing to be inoculated with anything from another person. He had reached the point where he could say that he did not know what causes leprosy.

Dr. Piffard could not understand why so many authorities regard leprosy as non-contagious, if they have read the report of the Royal College of Physicians upon this subject, and regard the writers of it as capable observers, and worthy of credence. From the time that he read that report, he was convinced that leprosy was most certainly capable of transmission in some way or other; not in the way scarlet fever is, perhaps, but in some way.

In 1843, a child belonging to British Guiana was inoculated by being vaccinated with matter from a leper; subsequently it came to this country, was received into Bellevue Hospital, and died there. Another case was subsequently admitted, and lived there two years, and died. Here were two cases which came in contact with hundreds of others. This occurred sixteen years ago—since then only two cases have been observed in the entire

State of New York. One of these had been associated with lepers in Mexico. The other was presented by Dr. Bulkley before the New York Pathological Society. It had never been outside of New York, and there were no assignable means of acquiring it from the former cases. These few cases show that there is very little danger of communicating leprosy in the ordinary way, like the ordinary contagious diseases, for instance.

The speaker claimed, as long ago as 1876, that this disease is inoculable, if the blood or secretions are brought into contact with a healthy person by inoculation, just like syphilis; and this is the usual means of transmission. These are the methods of communicating the disease, in which he understood Dr. White as agreeing with him.

Dr. White also believes the statement of Dr. Fisher, in charge of the hospital at Honolulu, who claims, in his report to the Board of Health of the Hawaiian Islands, that leprosy is only an old outgrowth from syphilis. At the meeting last week of the New York County Medical Society, the speaker took pains to combat the views of Dr. Fisher, at the request of the President of the Society. In preparing this communication, as a committee on the subject, he had sent circulars all over the country, to medical journals, and to members of this Association. This committee had a reply from only one of the members of the Association, Dr. Wigglesworth, of Boston, who said that he believed that there was a case in the hands of Dr. White. He had received a letter from physicians in Louisiana, and heard of some cases which turned out to be psoriasis. The committee therefore concluded that there must be only one case in this country!

Dr. Hardaway said that with regard to the nature of this disease we have evidence of the most contradictory sort, of reliable observers some pronounce it contagious, others are equally positive that it is not. When we learn the natural history of the disease better, we may learn that there are periods in which it is contagious, and at others not. It also appears to be hereditary just like syphilis. In some stages it may be contagious, in others hereditary. We may learn that at some times the disease may be active, and at others quiescent, and that it has distinct periods of development. In the course of time the virulence of the disease may wear out, and this may explain the fact that intimate connection with the disease fails to communicate it.

Dr. Duhring said that from reading reports of cases, his attention of late had been much more strongly drawn to the subject of the contagiousness of leprosy than before; there is a good deal of evidence pointing that way, but further than this he did not feel warranted in making any statement. The time has not yet arrived for making any positive decision. He asked with regard to the Cape Breton cases, if the reporter had included those of James Campbell and Neil McLean?

Dr. White said that he had.

Dr. Duhring stated that he had received a letter from Dr. Fletcher, asking his opinion upon these cases. He had been impressed with the careful manner in which the notes had been drawn up. He would not present this communication to the Association as nearly all of the information had been published. He believed that the main points are contained in Dr. White's paper.

Dr. Atkinson said that he only would express his unhesitating belief in the contagiousness of leprosy; he finds no obstacle in the slowness of development of the poison. There is almost a perfect analogy in syphilis. Slow development, impossibility of communication by atmospheric influence, or ordinary intercourse. We associate with syphilitic patients in the hospital, but do not acquire syphilis, and there is no contagious disease slower in its development, or more chronic in its course. He could understand why it is so little liable to communication, and most likely to be acquired by those in immediate intercourse with lepers.

Dr. Hyde said that he had not the same confidence that Dr. White had, that the contagiousness of leprosy is so definitely stated in the Hebrew Scriptures. The leper, it is true, was ceremonially unclean, but so was a woman while menstruating, or the man who had a gonorrhœal discharge, or a man who had touched a dead body. The leper passed along the street, crying, "unclean! unclean!" so that others might avoid him, not because he had a contagious disease, but in order that they might not be ceremonially defiled. We now know that psoriasis, scabies, and other skin diseases were confounded with leprosy, at that time. We have evidence of this in the cure of Gehazi, by Elijah. In referring to an art:cle by him upon this subject, the editor of the *Medical and Surgical Reporter*, of Philadelphia, said that he was wrong, but did not state why.

With regard to the bacillus lepræ, he had been much interested in Dr. Schmidt's paper which had been publishedd in the CHICAGO MEDICAL JOURNAL AND EXAMINER, and in the specimens also. He inquired if Dr. White had seen that paper.

Dr. White said that Dr. Schmidt had simply failed to find the bacillus lepræ.

Dr. Atkinson said that Dr. Bermann, of Baltimore, had asked Dr. Schmidt to send him some of his preparations, and some he had found full of the bacillus.

Dr. Hyde.— The paper of Dr. Schmidt and the sections, were sent by the author to a committee in Chicago, and were referred by the committee to Drs. Fenger, Curtis and himself. The great care and patience with which the preparations were made, was very evident. The speaker went over the specimens, and found in some a number of bacilli, but in others there were none. In every case in which these bacilli were found, there had been an open wound or the slide had been exposed to the air; in no single instance, in which a section of a tubercle had been kept from the external air, and carefully mounted, was there any bacillus lepræ to be found. He therefore concluded that it is a myth.

Dr. White said that Dr. Hyde must admit that he simply failed to find what others claim to have found. He had no doubt that Dr. Schmidt is a truthful and able observer, but no one would say that these other gentlemen were not the same. We are in the position of having one observer fail to find what many others of equal reputation have succeeded in finding. Koch and Cornil and others affirm the existence of the bacillus lepræ.

Dr. Atkinson did not consider it as a matter of chance, but one of skill and experience. In the specimens that he exhibited at Niagara Falls, carefully prepared by Dr. Bermann, these bacilli were shown perfectly. After reading Dr. Schmidt's communication in the CHICAGO MEDICAL JOURNAL AND EXAMINER, he examined a portion of a tubercle which he cut out of the lobe of the ear, which had not ulcerated. He was willing to stake his life on the fact, after an examination of this specimen, that in the tubercles of leprosy and the neighboring parts, there is a bacillus that is not due to exposure to the air, but is developed in the new growth. The finding of this bacillus is not a mere matter of putting a section under the microscope and looking at it, but of properly managing the light and the method of examination. The bacillus lepræ exists; whether it is the cause of the disease or not is another matter.

Dr. Rohé said that he was familiar with the paper of Dr. Schmidt. He could state that no one could know Dr. Schmidt personally, without being convinced that he never says anything that he does not absolutely believe to be true.

Dr. White, in conclusion, referred to the experience of one man, Prof. Koebner, who for three years examined cases of leprosy without finding any, but now finds bacilli in every one. What makes the difference? There is the same careful, conscientious man as before; but it is simply experience and skill. If we throw out these results, because a few are unable to obtain them, we should likewise throw out the result of the study of the minute anatomy of a great portion of the human frame, because there is still some difference of opinion with regard to it.

### EVENING SESSION.

Dr. C. Heitzmann, of New York, made some remarks on the use of ergot in skin diseases.

### DISCUSSION.

Dr. Robinson said that he saw no reason for changing the opinion he had previously expressed. A great many cases of acne had been benefited, but no cases had been cured. He believed that ergot could assist local remedies, as it seems to act upon the arterioles and capillaries, and perhaps more upon the muscular fibers of the arterioles and the erector pili muscles of the skin. It does reduce hyperæmia and diminish the number of acne spots, if not causing a complete disappearance of the eruption.

Dr. White inquired if ergot was used alone in the cases.

Dr. Heitzmann said that he agreed with Dr. Robinson that ergot only aids, and that it is necessary to the cure to use other remedies.

Dr. White asked what benefit is obtained from ergot more than from local remedies alone?

Dr. Heitzmann said that after using local remedies without success he had obtained a cure by administering ergot in addition. After using the ergot, the acceleration of the cure was great; he did not attempt to explain the cause.

Dr. White.—The physiological action of ergot upon the involuntary muscular fiber is known, and it has been used in many congestive affections; but it seems that it is just in acute hyperæmic disorders of the skin that Dr. Heitzmann has observed that it has no power; but in so ill-defined a disease as pruritus, and in so uncertain a disease as acne, he had found it to do the greatest good. The speaker had given ergot only in cases of pruritus, but he could not say that any of his cases had been benefited by it, and he could not understand how any one could say it. He could not see how it would be possible to determine this point; every case is a rule unto itself. The disease is a mild one and may terminate at any time; we can not positively say that we have abbreviated it a single day. Acne runs such an irregular course that he could not see how ergot could be of any good. He had a case of urticaria which he had been in the habit of showing to the class, to illustrate how many remedies may be given in urticaria. The case lasted nine weeks; the woman returned from an extended trip in the south, no better. The day after her return, the hottest day in the year, the eruption disappeared and has never returned. Had he given ergot as the last drug, the cure would have been attributed to it. Unless a large number of cases were presented, and especially a series in which the drug was administered alone, it could not be possible to arrive at any conclusion, especially with regard to such a disease as acne. He had considerable experience in the disorder, although he never gives internal treatment, and until statistics were presented showing success better than his own, he was not likely to change.

Dr. Piffard said that he had been present at the New York

Dermatological Society when Dr. Denslow read the paper referred to, and shortly afterwards presented a case before the Society which had lasted about a year and in which a number of remedies had been tried; it was a bad case. Dr. Denslow took her under his care; he gave her ergot and used mercurial ointment locally; the same ointment had been previously used without result. Shortly afterward Dr. D. left the city and she returned; and he had been very much impressed by the marked improvement. He left off the mercurial ointment, but kept up the ergot until she was completely cured for the time. The time went on, four months perhaps, and she returned with the same eruption on her face; she was given ergot and the face cleared again. Now when the disease comes back, she goes at once to the ergot without consulting him. This case can be taken as convincing as to the usefulness of ergot. In this way therapeutic experience is built up. In another case he had been obliged to discontinue arsenic on account of albuminuria; and in erythema and rosacea he had not seen it do good, but in cases where there was a doubt whether it was erythema nodosum or rosacea, it had always been serviceable.

In prurigo he had tried it, and in some cases where the crops came out from time to time; after the ergot was given no more crops appeared. In psoriasis he had given it because he had used a drug, *ustilago maidis*, in ichthyosis, a similar disease.

He was certainly very glad to hear the experience of Dr. Heitzmann, especially as he is usually skeptical about the effects of medicines given internally in cases of skin disease. Dr. White inquires, how can we tell whether the results obtained are the effects of the drug or not? In just the same manner as observed with regard to local remedies.

Dr. Robinson inquired as to the condition of the uterus in the cases benefited?

Dr. Piffard said that the uterine condition was bad; he sent her to Dr. Castle, who made an examination and treated her locally. The acne got well long before the uterus did; in fact the uterus is not cured yet.

Dr. Hardaway said that he had used ergot seven or eight years ago for a certain class of cases. He thought that he had seen it do good in erythematous eczema; and it is his habit to give it in large doses (3 drchms. fld. extract) in eczema, and believes it worthy of further trial. A number of cases have recovered apparently from the effects of the ergot; but he had used it combined with local measures. In the treatment of rosacea, the use of ergot by hypodermic injection is an old treatment; it was recommended at least ten years ago; he had used it locally in this disorder for a number of years, and also in eczema. He had been in the habit of combining twenty or thirty grains of ergotine with the diachylon ointment.

He had also treated urticaria with ergot, very chronic, inveterate cases, but had had no result whatever. In one case, after going through the Materia Medica without effect, he tried with ergot, but also with no results. This patient had also alopecia areata—a rare combination. He afterward took a sea voyage and returned well.

He had never been able to satisfy himself that he had obtained any result from ergot in purpura; he had also used it in peliosis rheumatica, without benefit. In acne he had used ergot; he had also used Vleminckx's solution locally with splendid results. He had seen results from this solution which he had not been able to obtain by other means. Possibly the good effects observed by Dr. Heitzmann, were due solely to the application of this solution.

Dr. Duhring inquired the number of cases of psoriasis in which it had been tried.

Dr. Heitzmann said only three or four.

Dr. White, in reply to a question, said that he never used specific remedies for acne. The great majority of cases need medicine internally, but it is not given for any specific action on the eruption.

Dr. Hyde had used ergot in purpura a number of times, and it had seemed perfectly valueless. He had several very interesting cases during last year and this year. He had used Squibb's ergot exclusively, but without effect. With regard to the theory of Dr. Denslow as to the state of the muscles in cases of acne, it seems that persons even with the most vigorous muscles in early life have acne, and in after life, when the muscles lose their tonicity, we see less.

Dr. Heitzmann said that he thought that what he had said was cautious enough. Dr. White is correct in saying that if we wish a thorough report, we must have a series of cases. This he would not deny; he simply had said that he had seen certain results in a number of cases, and that is all he intended to say. Truth conquers finally. We do not know how we cure a patient; we are only certain that we may cure them. His only desire was that the drug might have a further trial. We need not rely upon old world methods, for we are in this country at least greatly ahead as for as dermatology is concerned. If ergot is capable of assisting other remedies, as stated by Dr. Robinson, it is not without value in skin diseases.

Dr. White said that he had not criticised the remarks of the lecturer, but his method; he would not himself have brought such a report before this body. He would not, for instance, have given ergot in combination with other treatment, but would have given it alone before reporting its effects. Dr. Piffard's case is one to the point; we should have the effects of a remedy alone and not mixed up with other treatment. The report of Dr. Heitzmann would have been much more convincing if the drug had been given by itself.

Dr. I. E. Atkinson, of Baltimore, presented a communication on Syphiloderma Papulosum Circinatum.*

DISCUSSION.

Dr. Heitzmann simply expressed his pleasure at hearing this admirable paper. The only similar case that he knew of is that depicted in Kaposi's Atlas. He had not met with any case of the kind himself.

Dr. White reported two cases of the disease, both of which he had seen on the same day; one a man, the other a woman. Neither exhibited any pigmentation; they were probably in the early stage of the affection. Being submitted to the class at Harvard, they were pronounced without hesitation to be ringworm. They looked like typical cases of tinea circinata. Nothing could be more deceptive than these cases, but a closer examination revealed their syphilitic character. Pigmentation rarely accom-

* Published in the *Jour. of Cut. and Ven. Dis.*, Oct., 1882.

panied or followed this form of disease, in the cases he had seen. In one case he had much doubt about it, until he had submitted it to microscopic investigation; he would have been obliged to agree with the diagnosis of the class, if he had looked simply to the face-lesion, without further examination.

Dr. Atkinson referred to a skin disease in negroes; the speaker thought that the study of these disorders in the negro presents great difficulties. Syphilitic affections, especially, assume very peculiar forms. He inquired if the lecturer could always satisfy himself in his diagnosis, whether they are syphilitic or not?

Dr. Atkinson said that he generally could; the papular varieties especially. The papules are very often pointed with pus, which shows like a pearl through the dark skin. He had never seen it depicted with the absence of pigmentation at the point of the papule, but had seen it, however, where a little pus could be extruded by pressure.

Dr. Taylor inquired how many cases the lecturer had seen of the disease he had reported?

Dr. Atkinson said that he had reported five or six, and had a recollection of several others.

Dr. Taylor asked the size of the papules when the involution began in the center?

Dr. Atkinson. They were probably the size of a small pea, and a pigment deposit might follow, spreading from the center, very much like erythema nodosum.

Dr. Taylor asked what regions were affected?

Dr. Atkinson. On the face, especially, but he had seen one case in which the eruption was quite as copious as in the patient of Jullien, where the body was nearly covered with it. In the majority of cases, the only remarkable thing about the lesions is the concentric arrangement.

Dr. Taylor asked if he correctly understood the lecturer to say that the late tubercular syphilide has no tendency to this extension, like ringworm?

Dr. Atkinson said; yes, the late papular-circinate lesion is an open lesion.

Dr. Taylor said he thought that this is not always so; he regarded the experience of Dr. Atkinson as exceptional. The speaker had pursued this study for years, and thought that it is not the nature of the early papular syphiloderm to run this course, but the late papular syphilide There is a decided predilection for this form, and a decided preference for the face, knees and elbows. He could only himself recall six cases of the form of lesion described.

There are diagnostic points about these cases, in the face, which have not been spoken of, and may help: There is never any inflammation of the hair-follicles; sometimes the hair will fall out, but there is no appearance such as is seen in tinea tonsurans. When it comes on the body, on the chest, it does not cover the pubic region, as tinea does, and it also appears on the knees.

The experience of the lecturer is unusual. The lesion in the face the size of a half-dollar to a dollar, and pursuing this crescentic course, is rare; he could not recall a case in which he had ever seen such a patch run larger than a dollar. With reference to the discoloration, he called attention to this point, which he had noticed: that on the face the border of redness will remain for a long time, whereas on the body it fades out; a point connected with the circulation.

Dr. Rohé said that a colored woman came to his clinic in Baltimore with a very peculiar eruption over the ear. He examined her, but refrained from making a diagnosis. She returned within a few days He still could not classify the lesion under any form of skin disease that he knew of. He suspected syphilis, found cicatrices in her mouth, and then referred her to the venereal department, where it was pronounced to be a case of syphilis. This lesion presented almost the identical characters described by the lecturer. Papules small, and with small acuminated collections of pus at the apex of each papule. He could not diagnosticate it as any form of pure skin lesion, as he had not seen a case of this form of syphiloderm before; but this instance was recalled to his mind upon hearing Dr. Atkinson's remarks.

Dr. Hardaway said that those who had seen much of skin disease in negroes, would endorse the lecturer's observation, that there is a tendency to pustule formation, a pyogenic proclivity, in papular lesions.

Dr. Hyde said that he had not had much experience with skin diseases in negroes, but he had seen the peculiar type of eruption referred to, and it struck him as being the best description he had ever seen.

Dr. Atkinson said that it may be that in negroes this form of eruption is more common. He had seen six or eight cases—possibly a dozen ; two new cases he had seen in the last six months. In all was seen a very close resemblance to ringworm ; this resemblance is shown very graphically in the photographs taken from the cases.

Dr. Taylor said that he had seen syphilitic patches as large around as a goblet. It commences as a tubercle, and looks as if it were going to be extensive; but as it extends it becomes more superficial, and it grows redder. He regarded it as not an early lesion, but as tubercular at the beginning.

Dr. White said that in the case he had referred to, there was no tubercle, or even a papule, in the case; it looked more like erythema, as it was more superficial; in fact, its appearance was most like ringworm. There was no positive proof; in not one case could he get a good history of infection, but he regarded it as absolutely an early manifestation.

Dr. Atkinson said that the way he had found one case was, that it had been brought to him as a case of ringworm without any fungus.

Dr. Taylor said that Dr. White refers to what he had described as the erythematous syphilide. There are three grand varieties : erythema, papule, and tubercle; the latter belong to the late manifestations. He did not, however, mean to criticise the doctor's remarks. He inquired if there had been much infiltration in the cases ?

Dr. Atkinson said that there was a distinct ring of infiltration around the growth, like ringworm. In the cases of general eruption, it was very interesting to watch this feature; he could hardly convince himself that it was syphilis.

Dr. Taylor said that his object in asking the questions was to find out whether it would be necessary to devote a whole chapter to this lesion, or if it could be classed with roseola, papulæ, and tuberculæ.

Dr. White, of Boston, chairman of the committee on Statistics, read his annual report, which was adopted and referred for publication.

### An Experience in Skin Disease.

Dr. White related an experience in skin disease, in the treatment of corns and warts. A lady who had been under treatment for some time, without effect, for large warts upon the hands, was cured by the use of a mixture of salicylic acid, cannabis Indica and collodion. He had since used the compound with very good results; usually ordering half a drachm of the acid, ten grains of cannabis Indica, and one ounce of collodion.

Dr. Piffard said that this formula had been published by Dr. Train Green, of Pennsylvania, in the *Medical and Surgical Reporter*, some weeks ago. He had tried it himself, without seeing the slightest effect from it.

Dr. White said that in fifty cases, he had never seen it fail. It should be painted on in successive coats, at short intervals, until three or four coats are given. The next day, the corn or wart is easily scraped off.

Dr. Piffard said that he preferred the cautery.

Dr. Hardaway had passed the electrical needle through the base of the growth, in the case of warts, and caused them to disappear.

Dr. Piffard read the following communication from Dr. Sherwell, with the notes of a

### Case of Pellagra.

The enclosed is the history of a case in Dr. G. H. Atkinson's service, and under the care of A. H. Brown, resident L. I. C. Hospital. It is, in my opinion, undoubtedly a case of pellagra, from the rarity of which in this country, I am induced to send it to be included in the list of material at your meeting.

When first seen by me, toward the beginning of July, the eruption, only at that time present on the forehead, cheeks, nose and ears, was decidedly erythematous, but I was in doubt as to its diagnosis; whether severe erythema eczema, or erythematous lupus running an extremely rapid course. Have not seen the case many times; probably four in all. At the consultation previous to the last, could not give positive diagnosis, owing to the fact of the appearance of lesions over the extensor surfaces of the hands and feet, but decided on my last visit that it must be a case of pellagra, as above stated. The skin lesions, and symptoms generally, correspond exactly with those given by all authorities on the subject.

The history is almost necessarily imperfect, as the patient is apathetic in mood now, and was probably never very intelligent. The possibilities as to purpura, or erythematous diseases other than the one claimed, have been carefully considered, and as positively excluded. The eruption on the back is of a different kind from that on other portions of body and limbs, and, I think, results from stasis by recumbent position.

S. SHERWELL, M.D.

Tommaso Caprile, æt. twenty-five; Italian; seaman. Born in Rapallo, near Genoa. Never lived in any other part of the country; never saw or heard of any one about his home having any disease like his. Lived on corn part of the time, but never saw any mouldy or black corn. Never had any skin disease before this time. Spot came first on the forehead, about January, 1882, and extended over the face, hands and body. Patient is despondent much of the time. Has not been at home much of late.

Had no peculiar sensations previous to or accompanying the appearance of the eruption, and no itching or peculiar sensations are present now.

Patient has suppurating glands about the sides of face and neck. Temperature has gone as high as $105\frac{1}{2}°$ at different times.

This morning there are fissures, and surfaces from which blood flows slowly.

G. H. ATKINSON, M.D.,
Visiting Surgeon.

A. H. MOORE, M.D.,
House Surgeon.

### OFFICERS FOR 1882-3.

*President.*

DR. R. W. TAYLOR, of New York.

*Vice-Presidents.*

DR. I. E. ATKINSON, of Baltimore.

DR. A. R. ROBINSON, of New York.

*Secretary.*

DR. ARTHUR VAN HARLINGEN, of Philadelphia.

*Treasurer.*

DR. GEORGE H. ROHÉ, of Baltimore.

The next meeting will be held at Lake George, on *Wednesday, Thursday* and *Friday*, August 29, 30 and 31, 1883.

INDUCTION OF OFFICERS.

Dr. Hyde, before introducing the President-elect, said that upon assuming the duties of his responsible office, he had asked the coöperation and support of the members during his term of service; now, at the end of two years, he could say that he had abundantly received it, and returned his sincere thanks to the members for their aid and consideration. He would bespeak equal support for his successor, Dr. Taylor, who needed no introduction.

Dr. Taylor, upon taking his seat, thanked the Association for the honor conferred upon him, which was entirely unexpected. He could not state positively that this was the happiest moment of his life, but he fully appreciated the sentiments which actuated the members in their choice, and he was as happy, almost, as he could be. He accepted the duties of the office, and would try to discharge them as well as his predecessor had done, and begged for the same hearty co-operation on the part of the members.

---

THIRD DAY, SEPTEMBER 1.

A morning session was held, at which specimens were exhibited by Drs. Robinson and Heitzmann.

A business meeting was then held, at which the time and place of the next meeting were determined, and some private business transacted.

The session then adjourned.

